# Neutrophil necrosis and annexin 1 degradation associated with airway inflammation in lung transplant recipients with cystic fibrosis

**DOI:** 10.1186/1471-2466-12-44

**Published:** 2012-08-17

**Authors:** Francis H C Tsao, Zhuzai Xiang, Adnan Abbasi, Keith C Meyer

**Affiliations:** 1 Division of Pulmonary and Critical Care Medicine, Department of Medicine, Medical School, University of Wisconsin, K4/910 Clinical Science Center, 600 Highland Avenue, Madison, WI, 53792-9988, USA

**Keywords:** Annexin 1, Cystic fibrosis, Lung transplant, Necrosis, Neutrophil

## Abstract

**Background:**

Neutrophils sequestered in lower respiratory tract secretions in the inflamed lung may undergo apoptosis and/or necrosis and release toxic cellular contents that can injure airways or parenchyma. This study examined the viability of neutrophils retrieved from the proximal airways of lung transplant recipients with bacterial tracheobronchitis.

**Methods:**

Integrity and stability of intracellular proteins in neutrophils from proximal airways and peripheral blood from lung transplant recipients with bacterial tracheobronchitis were analyzed via Western blot analysis and determination of neutrophil viability by morphologic appearance and flow cytometry.

**Results:**

Neutrophils in tracheobronchial secretions from lung transplant recipients with cystic fibrosis who had normal chest radiographic imaging but bronchoscopic evidence of purulent tracheobronchitis post-transplant were necrotic and associated with degradation of intracellular protein annexin 1. The neutrophil influx was compartmentalized to large airways and not detected in peripheral bronchoalveolar airspaces sampled via bronchoalveolar lavage. Peripheral blood neutrophils from healthy subjects cultured *in vitro* demonstrated that annexin 1 degradation, particularly to a 33 kDa annexin 1 breakdown product (A1-BP), was associated with neutrophil necrosis, but not apoptosis. Although annexin 1 degradation was not specific to neutrophil necrosis, it was a sensitive marker of intracellular protein degradation associated with neutrophil necrosis. Annexin 1 degradation to 33 kDa A1-BP was not observed in peripheral blood neutrophils from healthy subjects, but annexin 1 appeared to be degraded in peripheral blood neutrophils of lung transplant recipients despite a normal morphologic appearance of these cells.

**Conclusions:**

Neutrophils were necrotic from the proximal airways of lung transplant recipients with bacterial tracheobronchitis, and this process may begin when neutrophils are still in the systemic circulation prior to sequestration in inflamed airways. Annexin 1 degradation to 33 kDa A1-BP may be useful as a sensitive marker to detect neutrophil necrosis.

## Background

Chronic lung disease in cystic fibrosis (CF) is characterized by bacterial infection and intense, neutrophil-dominated airway inflammation. The release of large amounts of neutrophil elastase by neutrophils as they undergo necrosis is thought to be a major cause of damage to epithelium and lung matrix that leads to diffuse bronchiectasis and bronchial obstruction [1-4]. Neutrophils usually undergo apoptosis (programmed cell death) after leaving the peripheral circulation and entering the lung [5]. When apoptosis proceeds in an orderly fashion, potentially injurious granular constituents, such as proteolytic enzymes and oxidant-generating enzymes, remain substantially sequestered. However, if neutrophils undergo necrosis and are not ingested by tissue macrophages in a timely fashion, toxic constituents including proteases can be released from necrotic cells in an unregulated manner [5]. Neutrophil necrosis is probably the primary cause of airway and lung damage in the intensely inflamed CF lung [6-9], but little is known as to why the recruited neutrophils undergo necrosis, and there is no simple method that can identify neutrophils undergoing necrosis.

Although necrotic neutrophils in the airways release abundant amounts of proteases including neutrophil elastase that can be measured in bronchoalveolar lavage (BAL) fluid (BALF) [10,11], it is not easy to determine whether BALF elastase is actively released by neutrophils or released from necrotic neutrophils. We previously observed that neutrophil elastase in BALF of CF patients readily cleaved a BALF 36 kDa protein annexin 1 [11] which is also abundant in circulating neutrophils and monocytes [12]. In this study we used neutrophil intracellular annexin 1 as a marker to determine whether neutrophil apoptosis and/or necrosis were associated with intense airway inflammation in lung transplant recipients with CF during episodes of bacterial tracheobronchitis.

## Methods

### Isolation of bronchoalveolar lavage and airspace cells

We obtained BALF from healthy volunteers, patients with CF and clinically stable lung transplant recipients with CF (N = 14) undergoing post-transplant routine surveillance bronchoscopy as previously described [11]. All lung transplant recipients were receiving routine post-transplant immunosuppression with a calcineurin inhibitor (cyclosporine A or tacrolimus), anti-proliferative agent (azathioprine or mycophenolate), and low-dose prednisone (5-10 mg daily). Bronchial secretions were also aspirated (and diluted with 5 to 10 volumes of normal saline) at the time that BAL was performed from the bilateral lung transplant recipients with CF, all of whom had purulent secretions in large, proximal airways due to bacterial tracheobronchitis, which was characterized by grossly purulent secretions in their proximal allograft airways combined with visualization of inflamed, edematous endobronchial mucosae at the time of bronchoscopy. All of the transplant recipients were subjected to surveillance bronchoscopy at least 6 months following transplant and had no evidence of rejection on transbronchial biopsies, nor did they have clinical evidence of bronchiolitis obliterans syndrome (BOS). *Pseudomonas aeruginosa* or *Staphylococcus aureus* were isolated from their proximal bronchial secretions, but bacterial cultures of BALF (which was performed from a wedge position in a segmental bronchus to sample distal bronchoalveolar secretions) did not show significant bacterial growth (all <1 × 10^3^ colony forming units per ml) or a significant influx of neutrophils on BAL differential cell counts (BAL neutrophil percentage was <5% in all subjects). Chest radiographs performed on the transplant recipients did not show any significant abnormalities, and standard BAL cultures and examination of lung biopsy specimens were negative for any other pathogens. Specimens from a subset of 6 CF lung transplant recipients were selected for annexin 1 analysis. All protocols were approved by the University of Wisconsin Institutional Review Board and informed consent was obtained from all subjects.

The BALF was filtered through two layers of loose sterile gauze into a 50 ml tube, then centrifuged at 1,200 rpm for 10 min at 4°C using a Beckman Model TJ-6 centrifuge. The cell-free BALF was stored at -70°C before use. The cell pellets were washed with 35 ml incomplete Hanks balanced salt solution (HBSS) and spun at 1,200 rpm at 4°C for 10 min and then suspended in 1-2 ml HBSS. Total and viable cells were counted using a hemocytometer after mixing an aliquot of cell suspension and trypan blue solution. An amount of 20,000 cells was used for each cytospin slide preparation and a Diff-Quik Stain Set (Dade Behring AG, Dudingen, Switzerland) was used to prepare the cells for morphological analysis. The rest of the cell suspension was spun at 1,200 rpm and the supernatant was discarded. Approximately 5 × 10^6^ cells were suspended in 100 μl lysis buffer (0.01 M Tris, 1 mM ethylenediamine tetraacetic acid, 5 mM 2-mercaptoethanol, 1% Igepal CA-630 nonionic detergent and 2 mM phenylmethylsulfonyl fluoride, pH 7.4). The cell lysates were then sonicated for 30 sec two times on ice using a Virsonic cell disrupter at 60-watt sonic energy for optimal recovery of annexin 1. The cell lysates were centrifuged at 10,000 rpm for 2 min; the supernatant was saved and stored at –70°C before use and the pellet was discarded.

### Isolation of neutrophils and monocytes from peripheral blood

Peripheral blood was collected from normal volunteers or lung transplant patients and processed for neutrophil and monocyte isolation within 20 min. A 5 ml aliquot of heparinized blood was layered onto 2 ml of neutrophil isolation media (PolymorphoprepTM, Axis-Shield PoC AS, Oslo, Norway) and centrifuged at 2,000 rpm for 16 min at 4°C. The top layer of plasma was removed; the middle monocyte layer was collected and mixed with 15 ml HBSS followed by centrifugation at 1,200 rpm for 10 min at 4°C. The supernatant was aspirated and the monocyte pellet was re-suspended in 1 ml cold HBSS and kept on ice for later use. The lower neutrophil layer was transferred into a 15 ml tube. An aliquot of cold 15 ml HBSS was added to the tube and cells were spun down at 1,200 rpm for 10 min at 4°C. The supernatant was aspirated and the pellet was re-suspended in 0.5 ml cold HBSS. The neutrophil suspension was mixed with 5 ml of 0.2% NaCl on ice with gentle inversion for 30 sec to remove residual red blood cells. Then 2.5 ml of 2.5% NaCl was added to the tube with gentle stirring. The cell suspension was made up to 15 ml with cold HBSS and centrifuged at 1,200 rpm for 10 min at 4°C. The supernatant was aspirated and the pellet was re-suspended in 10 ml cold HBSS followed by centrifuging at 1,200 rpm for 10 min at 4°C. The supernatant was aspirated and the neutrophil pellet was re-suspended in 1 ml cold HBSS. The 1 ml suspensions of monocytes and neutrophils were first used for cell count and cytospin as described above. After cell count and cytospin preparation, the monocyte and neutrophil suspensions were centrifuged at 1,200 rpm for 10 min at 4°C. The supernatant was discarded and the pellets were re-suspended in RPMI 1640 medium (BioWhittaker, Walkersville, MD) in a ratio of 5 × 10^6^ cells per 50 μl medium. A small portion of the cells (2 × 10^6^) were lysed in 100 μl lysis buffer as described above and the cell lysate was stored at –70°C for protein and Western blot analyses. The remaining neutrophils were used for flow cytometric and culture experiments described below.

### Cell culture of neutrophils

On average 30 × 10^6^ neutrophils were typically isolated from each 10 ml of blood from a healthy subject. Five × 10^6^ neutrophils in RPMI were seeded per well of a 6-well culture plate. The final volume of each well was brought to 1 ml with RPMI culture medium. The RPMI culture medium contained 5% heat-treated fetal calf serum (FCS), antibiotic-antimycotic (200 U/ml penicillin, 200 U/ml streptomycin, 500 ng/ml amphotericin), 2 mM glutamine and 10 mM HEPES (GibcoBRL). In some studies a specified amount of phorbol 12-myristate 13-acetate (PMA) (Sigma Chemical Co., St. Louis, MO) was added to the reaction mixture to test the effects of this substance on PMN viability and annexin 1 degradation. The plate was incubated in a 37°C 5% CO_2_ incubator for a specified time. After incubation, a rubber policeman was used to mobilize cells and the cell suspension was placed in a 10 ml tube. Each well was washed with 0.5 ml cold HBSS and the wash was combined with the original cell suspension and chilled on ice. Cells in each suspension were counted and cell viability was evaluated using the trypan blue exclusion method. An amount of 20,000 cells was withdrawn for one cytospin slide preparation; usually 6 slides were processed for samples from each well to perform morphological analysis. After cells were withdrawn for cytospin preparations, the cell suspension was centrifuged at 1,200 rpm for 10 min at 4°C. The supernatant was saved and stored at –70°C for protein assays as described below. The pellets were re-suspended in 5 ml cold HBSS and the suspension was centrifuged at 1,200 rpm for 10 min at 4°C. The supernatant was aspirated and the pellet was lysed in 100 μl lysis buffer followed by sonication as described above and the lysates were stored at –70°C for protein and Western blot analyses.

### Western blot analysis

The protein content in each sample of cell lysate was determined by the method of Lowry [13] modified for analysis in 96-well plates. Western blots were used for qualitative analysis of the proteins in the samples. A specified amount of protein (20-50 μg) in cell lysate was used for protein separation using a Bio-Rad Mini-PROTEAN 3 Cell Assembly Unit with the use of a 10% sodium dodecyl sulfate (SDS) Ready gel (Bio-Rad, Hercules, CA) under denaturing conditions. Proteins separated on the gel were then transferred onto a nitrocellulose membrane (pore size 0.45 μm) using a Bio-Rad Mini-Trans-Blot Electrophoretic Transfer Cell. The membrane was dried and soaked in 10 ml of TBST buffer (10 mM Tris-HCl, 150 mM NaCl, 0.05% Tween-20, pH 8.0) containing 5% dried non-fat milk and rocked for 30 min to block non-specific antibody binding sites. It was then incubated with guinea pig antiserum against rabbit lung annexin 1 (1:2000 dilution) in 10 ml of the same buffer overnight at room temperature. Previous studies demonstrated that the guinea pig anti-rabbit annexin antibody was highly specific for annexin 1 and cross-reacted with human annexin 1 [11]. The membrane was washed thoroughly in running distilled water and then rocked in 3 × 10 ml of milk-free TBST buffer with each rinse for 10 min. Then, the membrane was exposed to the second antibody of goat anti-guinea pig IgG conjugated with horse radish peroxidase (Sigma) (1:2000 dilution in 10 ml TBST) for 3 h. The membrane was thoroughly washed in running distilled water, then in 4 × 10 ml TBST buffer for 10 min for each washing. The immunoblotted protein was detected by the enhanced chemiluminescence (ECL) Western blotting analysis reagents followed by exposure of the membrane to a piece of high performance chemiluminescence film (Hyperfilm ECL, Amersham Pharmacia Biotech, Poscataway, NJ) according to the manufacturer’s procedures.

In some studies the annexin 1 blotted membrane was immersed in 10 ml of 0.5 N NaOH for 5 min at room temperature to strip off the annexin 1 blot followed by washing in distilled and deionized water and TBST buffer for 20 min. The TBST wash was repeated one more time. The membrane was then blotted with goat anti-human actin antibody (Santa Cruz Biotechnology, Santa Cruz, CA) (1:10,000 dilution) as the primary antibody and then treated with donkey anti-goat IgG conjugated with horse radish peroxidase (Santa Cruz Biotechnology) as the second antibody by the procedures described by the manufacturer. The actin signal was detected by the ECL method as described above.

### Flow cytometric analysis of neutrophil apoptosis and necrosis

Apoptosis of neutrophils was analyzed by double staining with annexin V-FITC and propidium iodide (PI) (BD Biosciences Pharmingen, San Jose, CA) and flow cytometry. To determine relative numbers of apoptotic vs. necrotic cells, 1 × 10^6^ neutrophils were suspended in 1 ml of 1x annexin V binding buffer. An aliquot of 0.1 ml cell suspension was combined with 5 μl of annexin V-FTIC and 5 μl of PI and the cell mixture was kept in the dark for 15 min at room temperature. The cells were then analyzed by flow cytometry within one hour.

## Results

### Morphological analysis of neutrophils

**Figure 1:**
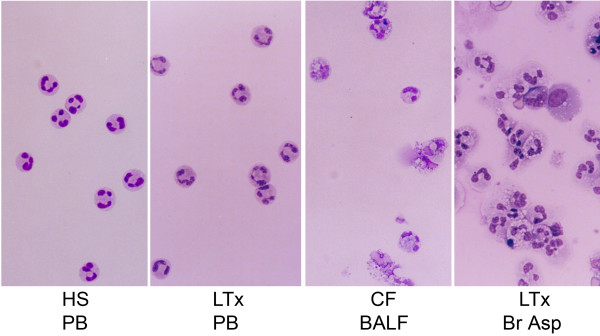
** Morphological analysis of neutrophils isolated from peripheral blood from a healthy subject (HS PB) and a lung transplant recipient PB (LTx PB), bronchoalveolar lavage fluid from an un-transplanted patient with CF and advanced bronchiectasis bronchoalveolar lavage fluid (CF BALF), or bronchial aspirate from a CF patient post-transplant (LTx Br Asp).** Cells were prepared on the cytospin slide, stained with Diff-Quik Stain and images photographed (Olympus BX60, 40X). The results are representative neutrophils from 3 HS, 3 CF and 6 LTx.

Peripheral blood neutrophils from patients with lung transplant recipients (LTx PB) appeared to be morphologically similar to those obtained from healthy volunteers (HS PB) (Figure 1). Most neutrophils isolated from BALF from non-transplanted subjects with CF (CF BALF) or from aspirated proximal airway secretions of lung transplant recipients (LTx Br Asp) whose allografts had bacterial tracheobronchitis appeared swollen and had vacuoles in their cytoplasm. Cell membrane disintegration was also observed in airspace neutrophils from CF BALF and LTx Br Asp. These changes were not observed in peripheral blood neutrophils taken from either healthy subjects (HS PB) or from patients with lung transplant recipients (LTx PB).

### Western blot analysis of BAL cells and peripheral blood cells

**Figure 2:**
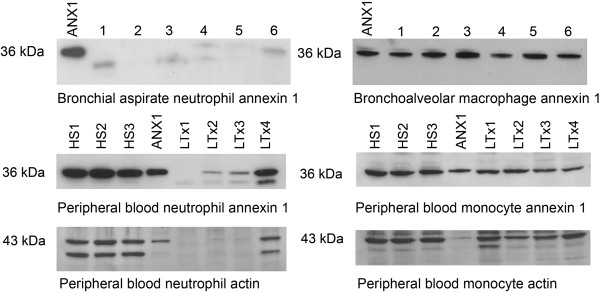
** Western blot analysis of proteins in BAL cells and peripheral blood cells.** Left top, neutrophils from bronchial aspirates from lung transplant recipients with CF and bronchial inflammation due to bacterial tracheobronchitis: ANX1 represents reference annexin 1 and lanes 1-6 neutrophil lysates of six patients. Left middle and bottom lanes of healthy subjects 1-3 (HS1-HS3) and lung transplant recipients (LTx1-LTx4) represent annexin 1 and actin, respectively in neutrophils. Right top, bronchoalveolar lavage fluid macrophages from lung transplant patients: and lanes 1-6 macrophage lysates of six patients. Right middle and bottom lanes of HS1-HS3 and LTx1-LTx4 represent annexin 1 and actin, respectively in monocyte. ANX1 represents reference annexin 1. A total of 50 μg protein in each cell lysate sample was used for Western blot analysis.

Alveolar macrophages were the most prevalent cells in BALF (> 95%) from healthy subjects or transplant recipients who did not have distal bronchoalveolar inflammation (neutrophils in BAL cell differential counts <4%). In contrast, when bronchial secretions were collected, neutrophils were always the most abundant cells (>90%) in the bronchial aspirates obtained from transplant recipients who had intense, proximal large airway inflammation. In the lysates of neutrophils from bronchial aspirates from lung transplant recipients, annexin 1 was nearly absent (Figure 2 left top lanes 1, 2, 3 and 5), in trace amounts (Figure 2 left top lanes 4, 6), or in trace amounts of 33 kDa annexin 1 breakdown product (A1-BP) (Figure 2 left top lanes 1, 3, 4). In comparison, annexin 1 was present and intact in BALF macrophages of six transplant recipients (Figure 2 right top lanes 1-6).

Annexin 1 was intact in the neutrophils and monocytes of healthy subjects (HS1-3) (Figure 2 left middle and right middle). However, annexin 1 in the neutrophils of three lung transplant patients was either absent, deficient (LTx1-3) or degraded to 33 kDa A1-BP (LTx4) (Figure 2 left middle). Contrarily, annexin 1 in the monocytes of these patients was intact, similarly to that of healthy subjects (Figure 2 right middle). Analysis of the conserved protein actin showed that actin was also deficient in neutrophils from the 3 LTx patients in which annexin 1 was deficient (Figure 2 left bottom). In monocytes the actin Western blot pattern of LTx patients was similar to that of HS (Figure 2 right bottom). The actin blot of neutrophil lysates showed that two protein bands, 43 kDa and 36 kDa were immunoblotted, but the predominant immunoblotted protein in monocyte lysates was 43 kDa actin. The anti-actin antibody might cross-react with an unknown 36 kDa protein. The anti-actin antibody did not recognize 36 kDa annexin 1, but it detected actin in annexin 1 controls (ANX1), which were prepared from rabbit lung cytosolic fractions [11] that contained actin (Figure 2 right and left bottom).

### Western blot, morphology and flow cytometry analyses of primary cultured peripheral blood neutrophils

**Figure 3:**
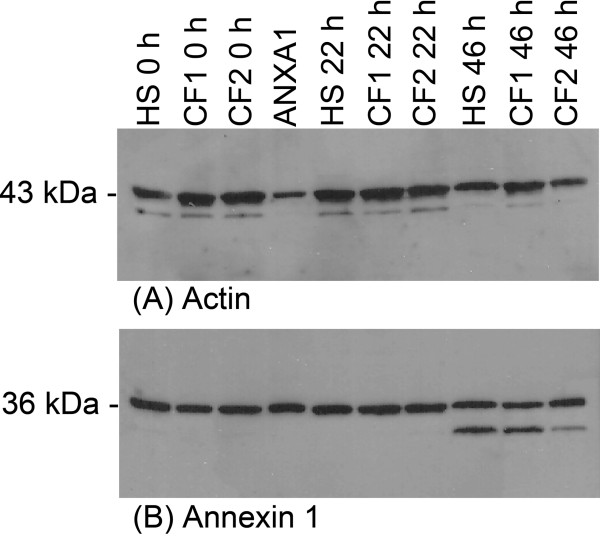
** Western blot of actin (A) and annexin 1 (B) in cultured peripheral blood neutrophils.** Peripheral blood neutrophils from healthy subjects (HS) and CF were cultured for 0, 22 and 46 h. A total of 50 μg protein in each cell lysate sample was used for Western blot analysis. Each label of (A) and (B) columns represent the same sample. Lane of ANX1 represents reference annexin 1.

Annexin 1 was intact in neutrophils freshly isolated from either healthy subjects or individuals with CF (non-transplanted) as well as in neutrophils cultured for up to 22 h (Figure 3B). After culture for 46 h, significantly increasing amounts of 33 kDa A1-BP were observed in both HS and CF neutrophils. The 43 kDa actin was also intact in neutrophils cultured for up to 22 h, but the amount of actin appeared to be decreased after 46 h culture (Figure 3A).

**Figure 4:**
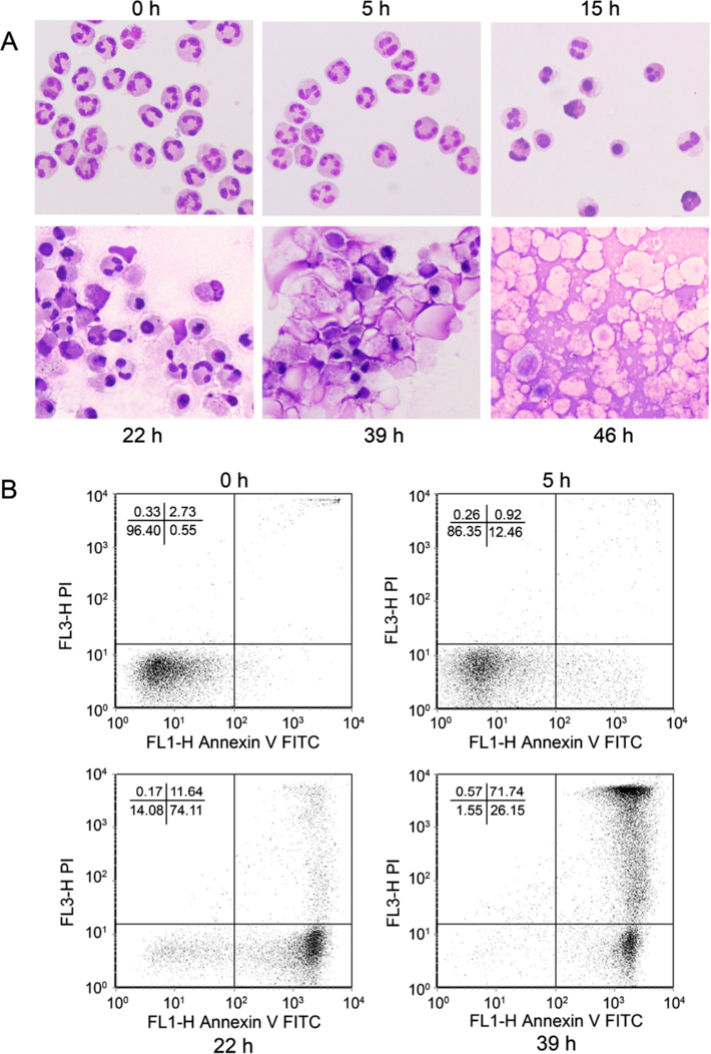
** Morphological and flow cytometric analysis of cultured peripheral blood neutrophils from healthy subject.** Neutrophils were cultured in vehicle medium for 0, 5 h, 15 h, 22 hr, 39 h and 46 h in RPMI culture media and cell morphology (**A**) and flow cytometry (**B**) were analyzed. Flow cytometric analysis could not be obtained for 46-h cultured cells because of clumping cells. Graphs represent annexin V-FITC and PI labeling. The lower left (LL) quadrant shows the percentage of viable cells. The lower right (LR) quadrant represents the percentage of early apoptotic cells labeled by annexin V-FITC. The upper left (UL) quadrant represents the percentage of dead cells labeled by PI. The upper right (UR) quadrant contains the percentage of advanced apoptotic and necrotic cells labeled by both annexin V-FITC and PI. The results are representative of 10 experiments. Morphological images were obtained by preparing cells on the cytospin slide, stained with Diff-Quik Stain and images photographed (Olympus BX60, 40X).

Morphological analysis showed that neutrophils exhibited distinct morphological changes during the period of culture (Figure 4A). Neutrophils had no apparent morphological changes after 5 h culture as compared to the freshly isolated neutrophils. However, after 15-22 h culture, condensation of cytoplasm and nuclei was clearly visible among the cells. Neutrophils cultured for 39-46 h showed enlarged, empty membrane vesicles with noticeable cell membrane disintegration.

Flow cytometry analysis of neutrophils showed that over 96% of freshly isolated neutrophils were negative to annexin V-FITC and PI staining (Figure 4B, 0 h lower left (LL) quadrant); the staining of annexin V-FITC (lower right (LR) quadrant) and PI (upper right (UR) quadrant) was less than 3% due to contamination of dead cells during cell isolation. After 5 h culture, positive annexin V-FITC staining increased to more than 12% (Figure 4B, 5 h LR), whereas PI staining was less than 1% (Figure 4B, 5 h UR). After 22 h culture, annexin V-FITC staining increased to 74% (LR); annexin V-FITC and PI staining increased to 12% (UR); viable cells were about 14% (LL) (Figure 4B, 22 h). After 39 h culture, viable cells were less than 2% (LL), annexin V-FITC stained cells were 26% (LR) and annexin V-FITC and PI stained cells increased to 72% (UR) (Figure 4B, 39 h).

### Effects of PMA on neutrophils

**Figure 5:**
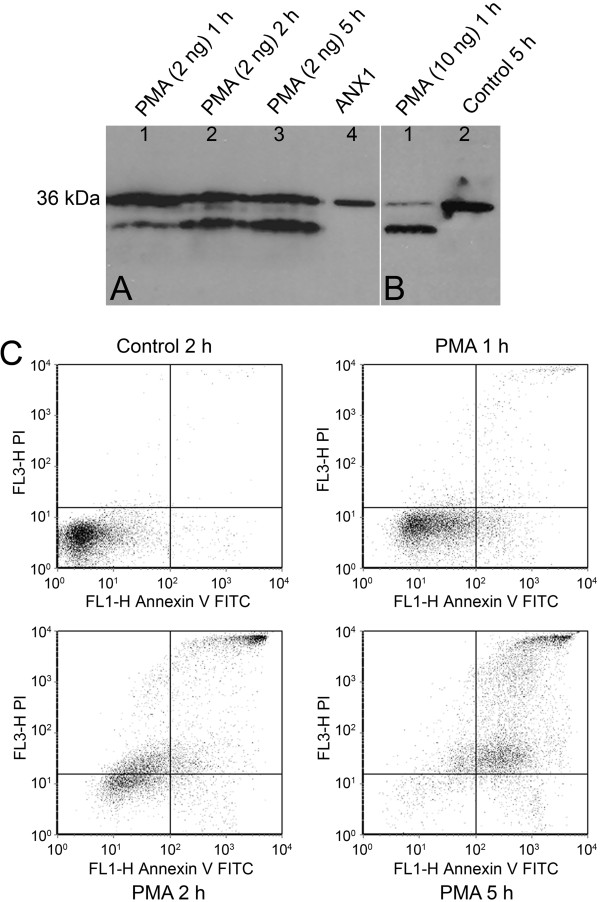
** Western blot and flow cytometric analysis of neutrophils treated with phorbol 12-myristate 13-acetate (PMA).** (**A**) Lanes 1 to 3 represent neutrophils treated with 2 ng/ml PMA for 1, 2 and 5 h, respectively. Lane 4 (ANX1) is reference annexin 1. (**B**) Lane 1 represents neutrophils treated with 10 ng/ml PMA for 1 h and lane 2 represents neutrophils cultured in vehicle medium for 5 h. Each neutrophil lysate used for Western blot analysis contained 50 μg protein. Neutrophils from the same culture of panel A were also analyzed by flow cytometry (**C**). The results are representative of 8 experiments.

The presence of 2 ng/ml of PMA in the culture medium noticeably induced the degradation of annexin 1 to 33 kDa A1-BP in neutrophils during 1 to 5 h incubation (Figure 5A lanes 1-3). Most annexin 1 was degraded to A1-BP in neutrophils in the presence of 10 ng/ml PMA in the culture media after only 1 hr incubation (Figure 5B lane 1). No annexin 1 degradation was observed in neutrophils cultured in vehicle medium even after 5 h incubation (Figure 5B lane 2). Trypan blue exclusion staining showed that neutrophils cultured in vehicle media were approximately 100% viable after 2 to 5 h incubation. In contrast, the viability of neutrophils exposed to 2 ng/ml PMA in culture for 1 hr was 95% and 60% for 2 h. Flow cytometric analysis showed that neutrophils exposed to PMA for 1-2 h in culture were rapidly stained by PI, but minimally by annexin V-FITC, if at all (Figure 5C). Morphological analysis also showed that PMA-exposed cells had membrane disintegration character of necrosis (data not shown).

## Discussion

Large numbers of neutrophils become sequestered in the airways of patients with CF in association with chronic bacterial infection [1-3,14] and neutrophils can also appear in relatively large numbers in bronchoalveolar secretions of lung transplant recipients with CF as well as those of recipients with other transplant indications as a consequence of reperfusion injury, infection, or delayed allograft dysfunction [10,15,16]. We observed that intracellular intact annexin 1 was largely deficient in neutrophils from bronchial aspirates of lung transplant patients with CF and bacterial tracheobronchitis. However, annexin I was abundant and intact in BALF macrophages. The neutrophils from bronchial aspirates were in advanced stages of apoptosis or necrosis as shown by morphological degeneration.

Annexin 1 degradation did not appear to be limited to neutrophils sequestered in the central airways from which the aspirates were obtained. It was also degraded to some extent in neutrophils from peripheral blood of these lung transplant recipients despite images that showed normal peripheral blood neutrophil morphology. Although annexin 1 degradation was not specific (actin was also significantly degraded in these peripheral blood neutrophils), annexin 1 degradation to 33 kDa A1-BP can be more readily detected in comparison to a decrease in neutrophil protein content. Extensive hydrolysis of annexin 1 and 33 kDa A1-BP may lead to relative depletion of this protein in neutrophils. It is unlikely that the degradation and deficiency of annexin 1 were due to sample processing, because annexin 1 and actin were intact in the lysates of HS peripheral blood neutrophils (despite the presence of abundant neutrophil proteases) and in macrophages. Also, no significant breakdown of annexin 1 and actin in monocytes was detected. Thus, degradation of intracellular annexin 1 in airspace neutrophils and peripheral blood neutrophils from these lung transplant recipients may have occurred intracellularly prior to *ex vivo* cell lysis and homogenization. We acknowledge that immunosuppressive or other medications that were taken by the lung transplant recipients may have affected neutrophil apoptosis or necrosis or annexin 1 degradation for the lung transplant recipients, and the effect of these drugs or other medications taken by these patients on neutrophils has not been examined.

A recent study has shown that the infection-related toxin, Panton-Valentine leukocidin (PVL), induced acute lung injury via necrotic neutrophils sequestered in alveoli [17]. Additionally, Watt et al. [18] found that patients with CF and *Pseudomonas aeruginosa* or *Burkholderia cenocepacia* infection had a significantly lower percentage of viable neutrophils and high levels of secondary necrotic granulocytes in sputa versus those from patients without Gram negative infection. Also, it has been shown that circulating neutrophils from triathletes subjected to heavy exercise show evidence of activation and DNA fragmentation, suggesting that apoptosis can occur in neutrophils while in the peripheral circulation [19]. Additionally, neutrophil elastase can be released from azurophilic granules and translocate to the cell nucleus when neutrophils are activated and synergistically drive chromatin decondensation with myeloperoxidase [20]. We speculate that degradation of annexin 1 and actin in the peripheral blood neutrophils of lung transplant patients might be due to proteases released during degenerative processes that occur during cellular necrosis. We also speculate that the impaired apoptosis of CF neutrophils observed by Moriceau et al. [21] may lead to a tendency of neutrophils to undergo necrosis. We have previously observed that peripheral blood neutrophils from patients with CF (not lung transplant recipients) more readily undergo apoptosis via TUNEL and flow cytometry assays (unpublished data). Additionally, we have found that bactericidal/permeability increasing (BPI) factor is greatly increased in BAL supernatant fluids from non-transplanted adults with CF (unpublished data), which likely reflects necrosis of airspace neutrophils and may provide a stimulus for the formation of autoantibodies against BPI [22,23]. Interestingly, we have frequently observed subclinical, proximal airway purulent tracheobronchitis (found at surveillance bronchoscopy in clinically stable patients) in bilateral lung transplant recipients with CF when chest radiographs show no evidence of lung infiltrates and BAL does not show neutrophilia or the presence of pathogens. For this reason, purulent fluid from proximal large airways (if present) is aspirated for gram smear and culture in addition to obtaining and analyzing BAL for evidence of infection. This phenomenon (purulent tracheobronchitis with unremarkable BAL analysis) is rarely observed in transplant recipients with non-CF lung disease as the indication for lung transplant.

It has been shown that neutrophils undergo spontaneous apoptosis under normal culture conditions [24], which is associated with nucleus condensation [25-27]. In this study we observed that most neutrophils from healthy subjects were apoptotic after culture for 15-22 h. There was no annexin 1 degradation in neutrophils after being cultured for 22 h. However, a significant amount of annexin 1 was degraded to yield 33 kDa A1-BP in neutrophils cultured for 46 h (A1-BP was also observed in 39 h-cultured neutrophils, data not shown). Neutrophils cultured for 39-46 h were found to be mostly necrotic (Figure 4). Although the amount of actin appears to be decreased in neutrophils after 46 h of cell culture, the actin change was not as striking as the presence of A1-BP.

PMA is known to induce morphological degeneration of neutrophils and cell death in culture [28,29]. We observed that in the presence of PMA, neutrophils in culture were rapidly labeled by PI within a short period of time. The path of neutrophil death induced by PMA appeared to differ from that followed by neutrophils undergoing apoptosis. We did not observe cell labeling with annexin V-FITC alone. Rapid labeling of PI was consistent with necrotic morphological degeneration of neutrophils (data not shown). In addition, neutrophils cultured with PMA exhibited rapid degradation of intracellular annexin 1 to yield the 33-kDa A1-BP. Annexin 1 degradation appears to be PMA dose-dependent and cell culture time-dependent, which suggested that annexin 1 degradation was associated with PMA-induced neutrophil necrosis.

## Conclusions

Neutrophils from aspirated proximal airway secretions and peripheral blood obtained from lung transplant recipients with CF had significant amounts of intracellular protein degradation, including annexin 1 and actin. Additionally, we found that bacterial infection and neutrophil influx can be compartmentalized to proximal large airways and absent in distal bronchoalveolar regions. Annexin 1 degradation to 33 kDa A1-BP in peripheral blood neutrophils might indicate that early stages of neutrophil necrosis have been initiated. Our findings suggest that annexin 1 degradation could be used as a sensitive marker to detect impaired apoptosis of neutrophils *in vivo* or in cell culture. Further research is needed to determine how activation and initiation of apoptosis in circulating neutrophils are triggered and whether this may promote or accentuate tissue inflammation and damage during emigration of these neutrophils from the microvasculature into inflamed tissues.

## Abbreviations

ANX1, Annexin 1; A1-BP, Annexin 1 breakdown product; BALF, Bronchoalveolar lavage fluid; Br Asp, Bronchial aspirate; CF, Cystic fibrosis; HS, Healthy subject; LTx, Lung transplant recipient; PB, Peripheral blood; PMA, Phorbol 12-myristate 13-acetate.

## Competing interests

The authors declare that they have no competing interests.

## Authors’ contributions

FT and ZX were involved in the study design, experiments and data analysis. AA was involved in the study design, sample preparation and data interpretation. KM was the PI and contributed to the study design, data interpretation and oversaw the project. FT and KM contributed to the preparation of the manuscript. All authors have read and approved the final manuscript.

## Pre-publication history

The pre-publication history for this paper can be accessed here:

http://www.biomedcentral.com/1471-2466/12/44/prepub
